# Medication management and practices in prison for people with mental health problems: a qualitative study

**DOI:** 10.1186/1752-4458-3-24

**Published:** 2009-10-20

**Authors:** Robert A Bowen, Anne Rogers, Jennifer Shaw

**Affiliations:** 1 Primary Care Research Group, School of Medicine, The University of Manchester, Manchester, UK; 2 Psychiatry, School of Medicine, The University of Manchester, Manchester, UK

## Abstract

**Background:**

Common mental health problems are prevalent in prison and the quality of prison health care provision for prisoners with mental health problems has been a focus of critical scrutiny. Currently, health policy aims to align and integrate prison health services and practices with those of the National Health Service (NHS). Medication management is a key aspect of treatment for patients with a mental health problem. The medication practices of patients and staff are therefore a key marker of the extent to which the health practices in prison settings equate with those of the NHS. The research reported here considers the influences on medication management during the early stages of custody and the impact it has on prisoners.

**Methods:**

The study employed a qualitative design incorporating semi-structured interviews with 39 prisoners and 71 staff at 4 prisons. Participant observation was carried out in key internal prison locations relevant to the management of vulnerable prisoners to support and inform the interview process. Thematic analysis of the interview data and interpretation of the observational field-notes were undertaken manually. Emergent themes included the impact that delays, changes to or the removal of medication have on prisoners on entry to prison, and the reasons that such events take place.

**Results and Discussion:**

Inmates accounts suggested that psychotropic medication was found a key and valued form of support for people with mental health problems entering custody. Existing regimes of medication and the autonomy to self-medicate established in the community are disrupted and curtailed by the dominant practices and prison routines for the taking of prescribed medication. The continuity of mental health care is undermined by the removal or alteration of existing medication practice and changes on entry to prison which exacerbate prisoners' anxiety and sense of helplessness. Prisoners with a dual diagnosis are likely to be doubly vulnerable because of inconsistencies in substance withdrawal management.

**Conclusion:**

Changes to medication management which accompany entry to prison appear to contribute to poor relationships with prison health staff, disrupts established self-medication practices, discourages patients from taking greater responsibility for their own conditions and detrimentally affects the mental health of many prisoners at a time when they are most vulnerable. Such practices are likely to inhibit the integration and normalisation of mental health management protocols in prison as compared with those operating in the wider community and may hinder progress towards improving the standard of mental health care available to prisoners suffering from mental disorder.

## Introduction

Mental health care provision in prisons constitutes an important system of mental health world wide. However, there has been long standing criticism of the care of prisoners with mental health problems and those at risk of self-harm and suicide [1]. Over the last decade a number of organisational and practical changes have been introduced with a view to reforming the system [1,2] with a particular emphasis on the impact of the early stages of custody. Measures which have been advocated and are gradually being implemented include increasing the availability of day care facilities to provide therapeutic settings in which members of community mental health teams (CMHTs) can run appropriate interventions, the expansion of wing-based in-reach services, the engagement of community-based health professionals to assist in promoting continuity of care on entry to prison and post-release, and self care [1,3]. The policy objective behind these changes has been predicated on the notion of equivalence in the range and quality of services available to prisoners and the integration and normalisation with NHS services. Expectations and assumptions behind this new approach include better recognition of the difficulties associated with adjusting to prison life, directing those finding it difficult to cope to appropriate psychological support, greater awareness of and identification of mental health problems, making appropriate referrals, and producing a care management plan (incorporating a medication regime if necessary) for those requiring care.

In spite of the increasing influence of NHS policy and practice, and a willingness to consider the broader determinants of prisoners' health, the notion that prisons can be supportive, healthy environments is at odds with the view that a therapeutic approach to mental health is undermined by an ethos that disempowers and deprives through processes devoted to discipline and control [4]. With estimates that as many as 95% of prisoners have a diagnosable mental health or substance misuse problem or both [2,5], the ability of prisoners to access primary care services and manage a mental health problem represents a basic indicator of the extent to which normalisation of NHS protocols and values may be judged to have been embedded in everyday Prison Service practice. Medication management is a key indicator of the extent to which prison mental health practices equate with those delivered in community settings. Whilst previous qualitative research has considered the factors influencing help-seeking for mental distress by offenders [6], the management and practices of managing medication has not been comprehensively explored. Amongst community populations previous research reports ambivalent attitudes to the taking and prescribing of medication. However, notwithstanding negative side effects, the taking of psychotropic medication for those living in ordinary community settings has been viewed as a key 'prop' in managing mental health. Additionally, shared decision making based on a concordance model which promotes the patients' active involvement has become an adopted norm within mainstream NHS provision [7]. Drawing on the narrative accounts of prisoners and the staff they must negotiate with, this paper considers the prescribing and taking of medication related to the management of mental health problems in a prison context.

## Methods

Ethical approval for the study was obtained from the South East Multi-Centre Research Ethics Committee. Data derived from a mixed qualitative methods approach incorporating semi-structured interviews that were supported and informed by participant observation was collected at 4 local prisons^1 ^[see Appendix 1] in England and Wales during 2004. The establishments comprised a female prison accepting all categories of prisoner (both sentenced and on remand) with facilities for juveniles and young offenders (YOs), a male YO and juvenile facility, a male Category B prison^2 ^[see Appendix 1] and a prison from the High Security Estate accommodating both remand and sentenced adults and YOs. At the time, all were undergoing an evaluated programme of structural and organisational changes intended to improve the management of prisoners believed to be at risk of suicide or self harm^3 ^[see Appendix 1].

A total of 71 members of staff and 39 prisoners were interviewed [see Table 1]. Members of staff were selected whose daily responsibilities brought them in contact with high-risk categories of prisoner (as described below). These 'key informants' included officers working in reception areas and on induction units, and health care professionals accustomed to managing high-risk patients. A purposive sample of prisoners was selected to provide 'information-rich cases for in-depth study' [8], and to enhance 'situational generalisability' [9] [See Table S1, Additional file 1 for further information]; these included prisoners who:-

**Table 1:** Demographic details of participants

**Prisoners**		
Gender	Male	27
	Female	12
Age	<25 years	13
	<35 years	17
	<45 years	7
	<55 years	2
Main offence	Drug related	6
	Acquisitive	12
	Violence	14
	Miscellaneous	7
Time in prison	<1 month	12
	<3 months	14
	<6 months	4
	≥ 6 months	9
Experience of F2052SH/ACCT	Yes	32
	No	7
History of mental illness	Yes	29
	No	10
History of self harm	Yes	27
	No	12
Drug/alcohol problem	Yes	25
	No	14
		
**Prison staff**		
Gender	Male	43
	Female	28
Role	Chaplain	3
	Detoxification staff	6
	Doctor	3
	Nurses/HCOs	16
	In-reach staff	8
	Social work/out-reach	2
	Prison officer	19
	Probation	1
	Psychiatry	4
	Psychology	1
	Suicide prevention coordinator	7
	Occupational therapist	1

1. were known to be suffering with or who had a recent history of mental disorder;

2. were currently withdrawing from drug or alcohol misuse;

3. had experience of either the F2052SH4 or ACCT5 processes (or both) [see Appendix 1];

4. had been in prison for at least 2 weeks and less than approximately 8 months.

### Semi -structured interviews

The interviews lasted approximately 45 minutes to 1 hour, and were recorded on a portable hand-held audio device using micro-cassettes.

Interviews with staff focused on participants' attitudes, and knowledge and training in relation to the identification and management of mental health problems. Staff were asked about their current practices, the division of labour and the impact that the environment had on mental health related work, and were asked about their professional relationships with other members of staff, and with the prisoners that they manage.

Interviews with prisoners explored participants' state of mental health on arrival in prison, their concerns at that time, and how these concerns were met. Prisoners were asked about the environment, regime and practices that they had experienced since entering prison, and the effect that these had had on their mental health. Prisoners were also asked to comment on their relationships with members of staff from various disciplines and their ability to access support networks.

A manual, iterative and reflexive approach to the thematic analysis of the interview data collected during this study involved the repeated review of both the audio recorded interviews and transcribed text to draw out key themes. Tables were then produced to highlight these issues; the tables permitting inter-group (i.e. between establishment/staff grouping e.g. nurses, officers, medical staff) and intra-group (i.e. between individuals within a particular establishment) comparisons to be made, assumptions derived that could be retested in the data collection process, and finally, conclusions drawn. This process closely follows that described by Miles and Huberman [10].

#### Participant observation

Structured observations intended to compliment the data accrued from the interviews centred on areas identified as having the greatest impact upon the detection and management of high-risk prisoners in a bid to capture representations of interaction between staff and prisoners. These areas included reception, induction, residential, in-patient and detoxification units. A non-participative approach with the intention of recording as much contextual information (through the noting of verbal comments, descriptions of the physical setting and details of associated processes and events) was adopted. Considerable importance was placed on efforts to merge with and cause minimal impact to the environment being studied. Periods of observation lasted between 2 and 7 hours in each location. Field notes were recorded using pen and paper. Analysis through the interpretation of observed events, consideration of alternative perspectives and meanings, and development of theory was initiated as the observations took place as suggested by May [11]; clarification of recorded events being sought through timely informal query or in the course of interviews with participants. Subsequent to data collection, field-notes were subject to review and re-evaluation with a view to further interpretation and comparison with interview data where possible.

## Results

The key themes to emerge from the data included the consequences that disruption to prisoners' medication on entry to prison had on their well-being, the impact of inherent restrictions within prison regimes and practices, illustrations of the ways in which prisoner-patients' autonomy in relation to taking medication is curtailed, the ensuing distancing of relationships between healthcare professionals and prisoners, and problems associated with dealing with comorbidity.

### Disruption to medication management: a barrier to coping with mental health and managing in prison

Sixteen of the 36 inmates suffering with conditions that included schizophrenia, bi-polar disorder, depression or anxiety expressed grievance about the medication regime that was imposed on entry into prison and the impact that this had on their mental state. The following quotes record the sense of concern experienced by prisoners who, on arriving in prison (some for the first time) were confronted with the realisation that they would have to cope without long-standing medication.

"I was on tablets for depression running back over the past 10 years, and when I came here, they refused to give me any...... so for just short of a month of being here, I didn't get any... And when I first came in and I explained it, I explained what medication I was on on the outside, and the doctor says 'well we don't give that out in here'. When he said " we don't give that out in here', I thought 'Whohh! That's what I've always had....'. They were listening but they weren't understanding....... That's how they are in here..... They've got their opinion in their head and nothing's gonna change that."

(male prisoner, ID 39)

The altering of medication without negotiation also created distress as recounted by the following participant who shortly before entering prison had been treated in a hospital psychiatric unit for bi-polar disorder.

*"I felt I was coping alright with these tablets and then when I knew I wasn't getting any, I just panicked really. The first night I was crying and.. I was beside myself really... because when I was in the hospital, I was on Trazodone (anti-depressant), and they [i.e. specifically the prison doctor] changed it to Venlafaxine. ...and that one I've forgot the name of, for the bi-polar, they just stopped them... It's quite a puzzle to me, 'cos I did get better in there [when previously in hospital], and I can't imagine how I'm going to be alright without it ...." ***(female prisoner, ID 9)**

Delays in getting access to medication that had been taken on a regular basis (in some cases prescribed during a previous recent stay in prison) were reported to result in a deterioration of individuals' mental states with the ensuing need to incorporate heightened levels of supervision or invoke what was perceived by prisoners to be punitive surveillance as illustrated by the following accounts:

*"I expected to [i.e. to receive medication], but I didn't take any for......until the end of the weekend..... [for] 4 days...' cos they didn't have any in the pharmacy..... I started going a bit mad, a bit loopy.. [I] self-harmed....And I asked them to put me on 2052, cos I didn't feel well." ***(male prisoner, ID6)**

*"The doctor told me he wasn't going to give me me anti-depressant ........So I said, all I said was ' it's no wonder people hang their selves'. It was taken the wrong way and I was taken to hospital and put in a 'strip cell' because they thought I'd said that I was going to hang meself.... I tried to explain that I'd only said it out of frustration because I mean, it is a worry. The medication does help. I've tried just about every anti-depressant. I've been on this one for than 3 years now." ***(male prisoner, ID4)**

### Discontinuity between medication in the community and prison: the importance of entry processes

In raising the issue of disruption of prisoner medication on entry to prison, several healthcare professionals who were interviewed cited a number of causes and effects that were felt to contribute to recognised inconsistencies in prescribing practice. Whilst some respondents noted the propensity of some prisoners to lie, a fundamental cause for the lack of continuity in receiving medication on entry to prison was attributed by one in-reach worker to the difficulty of ensuring that new reception prisoners with the greatest needs are seen promptly by the prison doctor. This participant stated:

"The only way really around it is that you need to revamp the system of people being reviewed [on arrival in prison]. If you can imagine, the courts sit 'til 5 o'clock. If someone is remanded, they mightn't get to the prison 'til 8 o'clock, 9 o'clock that night. They're [the nursing staff on duty] not going to start ringing GPs at that time of night. In which case, they're then referred to healthcare. If they're lucky, they'll see them the next day. If there's a huge number of people to be seen, they might not be seen for 2 or 3 days. These are where the delays occur."

The same member of staff also offered an explanation as to why some prisoners' requests to have what they claim has previously been prescribed for them by their community GP (general practitioner) continued in prison, would often be met with a firm refusal:

*"Where you get the problems is where someone comes in who is clearly going to need a detox also, who immediately starts to tell you that he's been taking Valium and Temazepam, and they've all been prescribed by his GP. You know.. of course they are [sarcasm inferred]. And the number of people that they [i.e. staff] do checks on, and they're not. They've [the prisoner] been buying drugs or whatever. So people tend to be less enthusiastic, shall we say, about making the phone calls and whatever, and just say to people 'I'm sorry, these drugs are just not available in this prison', which is not always correct... Valium is the obvious one. We can use Valium in the prison but it is extremely rare that we use it and it is a 'no-no'. Technically, in here, [it's] a non-formulary item, so you have to fill out another form. You have to get another doctor to agree with you so as to prescribe it, which is time consuming. So 99.9% of the time, they'll just tell you 'it's not available'..." ***(member of in-reach team^6 ^[see Appendix 1], ID 60)**

The problem of confirming prisoners' claims of having previously received prescribed medication was expanded upon by a nurse:

"...If they come in with drugs that are in their name, have pharmacy labels on them, then they get prescribed you see. But because they don't turn up with any evidence of what they've been taking, it is the problem of checking out with the GP surgeries, who are extremely reluctant I have to say, to give us information of what these guys are taking, so that we can continue that. Unless it was wildly outside the formulary which we adhere to, which is the SSSS formulary [the formulary drawn up by the local Primary Care Trust], we wouldn't be changing it, so there is some protection..."

(member of nursing staff, ID 49)

One further medication/treatment related issue that emerged in interviews with health care staff was the chaotic state of paper-based prisoners' medical records. The following comments refer to what was for each establishment, the eagerly anticipated link up with the NHS computer driven patient data system:

*"I would say that General Practice in here [in prison] is at about 1980 in terms of comparison with the outside world. The biggest deficit now is the lack of an IT system, an integrated IT system, which means we work entirely off paper notes, and have all the problems of paper notes which are that they are a mess, they are difficult to get information from them quickly... We can't trace back what drugs they've been on without having to trawl through the whole lot. ... Like, all the repeat prescribing has to be hand-written, hand-checked. ... We are really back to where I came into General Practice in 1980. However, we are supposed to have a reasonable computer system up and running by Easter, so hopefully when that all gets on then things like Clinical Governance, chasing through repeat prescriptions, monitoring, will all become a lot easier"*.

(doctor, ID 66)

### Curtailing autonomy and control over self-medication practices

The normative routines and practices employed by prisoners to manage their symptoms prior to entering prison were reported to be extremely limited once incarcerated. Prisoners noted the consequences of the perceived lack of flexibility in the prison regime and the limited availability of in-possession medication:

"I only had been taking the Trazodone of a night time [i.e. prior to coming into prison]. I had problems for quite a few weeks [i.e. after entering prison]. I used to get the tablet at 4 o'clock before tea at 5o'clock, and if I took the tablet at 4 [o'clock], by the time I come to 5 [o'clock] I couldn't even get myself off the bed because I was that drugged up on it.... But I've manage to get that moved to 7 o'clock now after a lot of negotiation."

(male prisoner, ID 18)

Another prisoner who reported spending the majority of his teenage years and adult life in care or penal institutions, and who had a long history of schizophrenia, contrasted the medication protocols and perceived efficiency of the healthcare service of other establishments with those of the prison in which he was currently residing. When asked if he was feeling the benefit of a change to his medication (the dose having recently been increased by the prison doctor in response to a deterioration in his behaviour which the participant believed had resulted from the prison psychiatrist having inappropriately reduced it on entering prison). The interviewee replied:

*"Not at the minute, no, 'cos Healthcare keep messing it up...Well they keep.. not bringing it to me. Not giving it to me...Well we'll see, 'cos I got my medication at 12 o'clock last night...I've been in about 3 weeks and it's happened about 5 times. So we'll have to wait and see what time it comes this afternoon." ***(male prisoner, ID 20)**

The outcome and veracity of this participant's comment was supported by the following observational record:

*Shortly after completing the interview with the previous participant i.e. male prisoner ID 20, I was observing the activity on the residential unit just as the afternoon medication round was being completed. It became apparent that the prisoner to whom I had just been speaking had been to see the nurse and had once again found that his medication was unavailable. This resulted in the participant and the wing staff immediately becoming engaged in a heated discussion. The manner in which he was pacing aggressively up and down the landing, and shouting at staff led to the conclusion that there was a strong possibility that he would be reprimanded or restrained for what was clearly angry behaviour borne out of his frustration. I was unable to view the outcome of this tense situation as my escort was ready to leave the house unit before the situation was resolved*. **Observation 6, Prison X, Friday 4 pm**

When medication was received, the lack of personal control over taking it was more curtailed than prisoners were used to, causing disruption to practiced means of controlling their symptoms. Healthcare staff recognised the benefits of providing some in-possession medication but were keen to point out the security and welfare concerns associated with certain drugs being used as currency, and the potential for suicidal prisoners to stockpile supplies. Whilst the suggestion that dispensing medication from the prison pharmacy or on the wings provided useful opportunities for monitoring patients' state of health, this approach was recognised as doing little to develop personal responsibility and was widely recognised as failing to meet many prisoners' needs with respect to the timing that medication should be taken. One doctor noted:

*"...If I write up a drug [i.e. a prescription for a prisoner] for three times a day, this is one of the issues that we are trying to deal with at the moment, they are going to get 3 doses, some of them, within as little as 8 hours. Whereas again, if you were at home you'd take them breakfast time, lunchtime and evening time, but because of the needs of the discipline staff to be monitoring the queues and things, then our medication regimes have to fit in with them, and it does lead to some friction. We are trying to work on that at the moment"*. **(doctor, ID 66)**

### Alienation and mutual distrust: anti-therapeutic relationships between staff and inmates over medication prescribing

Patient-centredness is a hallmark of high quality primary care within the NHS. In recent years a focus on negotiating medication with patients has become mainstream in primary and secondary care and the notion of a therapeutic alliance over medication and the provision of information has become normative. A majority of the respondents made it clear that they felt there was little point in speaking to the doctor as their requests to have their medication adjusted were generally ignored. One female prisoner who had a history of schizophrenia went further, stating that she was reluctant to engage with medical staff out of fear that complaining might result in her current medication (which had been prescribed by the doctor at the previous prison from where she had recently been transferred) being removed altogether. When asked if she queried the dose that she had been given, she replied:

"I didn't bother. I was more concerned about taking my medication, and I didn't want to say anything 'cos I thought if I said anything they might just take me off it... So I just kept my mouth shut basically. I daren't say anything. You know what they're like."

(female prisoner, ID 17)

The perceived arbitrariness of prescribing practice was central to the frustration and heightened anxiety experienced by many prisoners, and was identified as contributing to the strained relationships between inmates and healthcare professionals - a point illustrated by a participant who remarked:

*"Yeah.. with prison and the 'out' [outside community], it's different. Like, on the out, your doctor knows who you are, what you are, what medication you're on and what your problem is. In here, it doesn't matter what medication you're on out there, you don't get it in here. Do you know what I mean?" ***(male prisoner, ID 20)**

Whilst this participant's comment makes indirect reference to the benefits that follow from there being a history of contact with one's GP - an association that rarely develops during comparatively short stays in local prisons, one nurse alluded to the comparative ease with which difficult and potentially unpopular decisions regarding patients' treatment regimes could be implemented in prison settings, stating:

*"The standard of care [medical care] is good, and I would think that some of the inmates would think it was good, but a lot of them would think it was bad because they're not getting what they get on the 'out'... If a doctor is in his surgery on a little estate somewhere and someone comes in screaming and shouting for something, and he feels intimidated and wants his surgery to be nice and quiet, he'll give them a script, a prescription, and he's got them out the door.... But if you're in a place like a prison, where they can't go anywhere, they can't be disruptive or if they are disruptive, they can be removed, then you can say 'no, I'm not going to give you that drug'. And so I think that the general consensus might be that we've got rubbish doctors because 'the doctor on the 'out' would give me it'. But it doesn't necessarily mean that the doctor on the 'out' is good, it's not his fault but a lot of people get pacified on the 'out'. People get kept on Valium for years and it shouldn't happen"*. **(nurse, ID 8)**

Despite the level of concern expressed by prisoner participants in relation to their medication being changed, reduced or stopped completely, few health care staff made direct reference to these issues, and fewer still commented on the effects that such occurrences might cause. However, a prevalent theme among those that did contribute to this issue highlighted their concerns that prisoners were often disingenuous in their claims that they had been receiving some prescription drugs, as illustrated by the following quote:

*"Like there's one guy at the moment who is convinced that he's on certain doses of certain things and I've got the GP to read me his psychiatrist's letter that came in January, so I know that the doses we've prescribed are correct. Do you know what I mean? 'Cos I've seen him three times with the same issue... So there's a bit of that, and a bit of manipulation..." ***(member of nursing staff, ID 49)**

The suspicion contained within the previous participant's dialogue was supported in the frank opinion of a psychiatrist:

*"I think the big difference between civilian psychiatric practice and working here is that in civilian psychiatric practice people rarely actually lie to you. I mean, they highlight things they want you to be aware of and minimise things they don't want you to be aware of. I suppose it's lying really, but usually there's a kernel of truth in 95% of cases; whereas in here, 95% of the people that you're speaking to are telling you things that aren't true. That's a politically incorrectly explanation but ... The aim usually is to obtain either pain killers or opiates such as Co-codamol, just to get some kind of sedative so that they can basically blot out reality really... It's quite crucially important really [to understand what is going on] 'cos what happens is that if the doctors who are involved just give in when they [the prisoners] come in and start ranting and raving about opiates and so on, and the doctor kind of goes 'okay' and gives in to them then it makes it harder for the prison staff 'cos he goes back and tells the wing that Dr X is a walkover and then they are all coming over, and if they get codeine out of the doctor, they sell it for 'gear' [i.e. drugs] to other prisoners and it makes a breakdown of the system more likely." ***(psychiatrist, ID 26)**

Evidence of the dishonesty employed by prisoners and the potential for confrontation were recorded in the following extract of an observation of a health screening interview conducted with a newly arrived prisoner on the reception unit of one of the prisons:

*'...Throughout the interview, the prisoner appeared upset, avoided eye contact (looking down at the table much of the time), telling the nurse that s/he was 'rattling' i.e. suffering from the withdrawal from drugs, and couldn't be bothered answering the nurse's questions, other than to say that s/he had previously seen a psychiatrist but was not willing to say what for, or when. The prisoner then asked for his/her own medication that s/he had brought from court. This was refused, the nurse explaining that s/he would need to be seen by the doctor first. This was met by abuse - the prisoner shouting that s/he wanted them now... Approximately 10 minutes later, the prisoner was observed sat at a desk in reception talking to an officer who was noting personal details. The prisoner was seen to be relaxed, chatting calmly and joking with an officer*.

*Two hours later, the prisoner, returned to the reception unit to see the doctor as s/he came on duty. In the interview room, the prisoner immediately re-adopted the demeanour s/he had demonstrated when s/he had seen the nurse. Clutching a handkerchief to his/her mouth, his/her hands and legs were seen to shake. The prisoner avoided eye contact and responded to questions by a nod or shake of the head. The doctor focused on his/her current medication (which was recovered from her possessions) and the prisoner's drug problem. The doctor queried the prisoner's use of Amitriptyline, and s/he admitted that this was not for depression but more to help him/her sleep. The doctor then explained how a Methadone detox would be given but that it would start at 10 mg, rising to 30 mg and then tailing off. At this point the prisoner was very quiet. The doctor also explained that Diazepam would be given but it would be administered in 15 mg doses, one in the morning and one at night. Realising that the total quantity was much less that s/he had been used to, the prisoner remonstrated with the doctor. The prisoner was also told that although s/he would be given Amitriptyline that night, it would be reviewed by the doctor the following day. At this point, the prisoner became more agitated. The interview ended with the prisoner being led away, clearly disgruntled'*.

Observation 5 - Prison Y, approximately 16.30 hrs

### Dealing with co-morbidity and managing withdrawal from drugs

Twenty-five of the prisoners interviewed recorded a past history of drug or alcohol abuse. Although not everyone entering prison either requires or requests assistance with withdrawal, those who test positive to having an ongoing substance misuse problem are invariably offered a chemical detox. Participants' comments were found to focus on several areas of concern. Most significant was the variation in practice adopted by different prisons. The following prisoner's comments relating to the prescription of pain relief during detoxification typify the experience of many others:

*"I only started getting them 3 days after I came in. I had to wait for my medical records from GGGG [name of prison from where the prisoner had just been transferred] and until that came they couldn't give us any medication. Thing is, I'd been on Methadone there...Yeah, it's different in different gaols.... Like in GGGG, if you're on a script on the 'out', they give you what they call a 'maintenance script' inside, of a smaller dose. Whereas I was on 50 ml on the outside, so in GGGG I was getting 30 ml of methadone and a sleeping tablet. And that was it. That was doing it. But when I came here, they told me they don't do Methadone ..., they don't give you sleeping pills. It's a total no-no. So I was ill, very ill." ***(female prisoner, ID 15)**

Being moved from one prison to another, either for permanent transfer or in order to facilitate an appearance at court in relation to offences committed elsewhere around the country was once again found to cause disruption to prisoners' medication. One such example emerged in the comments of a participant who had been started on a course of pain relief to help with his detox from heroin when he first arrived in prison. He stated:

"... but when they shipped me from here to PPPP [a prison nearer to court], my detox medication, I never got that for three days."

The same participant went on to recall how during the 3 weeks he was at the other prison, he received medication to try to stabilise his mental condition. However, by the time he returned to the original establishment, although he had completed his detox, his mental condition deteriorated, causing him to be placed on the prison's in-patient unit for 2 days. The following comment once again highlights the lack of support felt by the interviewee from residential unit officers at a time when he was evidently feeling unstable, and highlights the number of different residential wings to which he had been assigned during the first 6 - 8 weeks that he had been in custody. He recalled:

*"... I approached a member of staff and told him I was still feeling a bit dodgy, mentally. And they didn't want to deal with the issues, they just shipped me straight off to another wing. And then I went, approached the staff on the other wing, told them, and they kicked me off that wing, they didn't want to deal with it, put me back on five [residential unit 5]. And then five put me on here [residential unit 3]." ***(male prisoner, ID 37)**

One other area of complaint to emerge in prisoners' dialogue relates to the absence of information provided by health care staff concerning the medication that they were given. Some prisoners clearly felt they were being patronised and their legitimate interest in the drugs they were being given was being disregarded by some health care staff. Whilst this approach on the part of some health care professionals may result from a wish to avoid confrontation or genuinely result from the view that the treatment being suggested is perceived to be in the patient's best interest, it belies the extensive pharmacopoeic knowledge that many prisoners who have struggled with enduring mental illness or substance misuse are likely to have, and demonstrates not only a lack of appreciation for the need for effective communication but appears to depart significantly from what would be considered good practice in the wider community. One prisoner described a conversation that took place during the health-screen interview. He recalled:

*"...this time when I came in I said 'I've got a heroin habit'. [The doctor] Said 'right, you'll be doing a Subutex detox'. 'Fine'. And I said to the doctor 'I've been on the crack as well'. And he went 'Right, well take five of these green pills'. I said 'what are they? I like to know what they are'. He said 'they're happy pills, they'll make you feel better'. And that was it, and I was told to go into the waiting room again." ***(male prisoner, ID 26)**

A major concern of staff involved in the day-to-day management of prisoners withdrawing from substances misuse echoed that of prisoners in so far as inconsistency in prescribed treatment regimes was associated with increased confrontation. One Health Care Officer (HCO - prison officer who has undergone some level of nursing training) stated:

*"I think it's a good thing as well that we're getting a dedicated detox unit. Er.. the thing is with the doctors we've used, the [GP] practice we had before and the [GP] practice we've got now, the detox is too erratic. You know, one doctor will give the 9-day detox, another will cut it down to 3 days or something... It doesn't work...I mean, it's very confrontational at the treatment room a lot of the time.... And there was like transfers [from another prison] in yesterday. ... And they all came up on Methadone detoxs. And we don't use Methadone here so consequently they went from a standard Methadone detox of 25 mg a day to the year dot [sarcasm inferred], to a 9-day liquid DF detox here and then that's it. So they were all creating hell last night when they came in"*. **(HCO^7 ^[see Appendix 1], ID 32)**

Equally important however, from a staff perspective, was the need to establish a practicable protocol for dealing with dual-diagnosis clients; the comments of in-reach and detox. staff indicating how creating a clear understanding of the division of labour and developing effective lines of communication (cornerstones of inter-disciplinary working) were essential in ensuring that available resources were appropriately tasked to support patients appropriately. The following quote describes how newly arrived prisoners, recognised by reception health staff to have a mental health and substance abuse issues, might be referred to various support services:

*"Well they tend to refer to.. like if someone has drug problems, mental health problems and self-harm, they will refer to us [detox], to CMHT [Community Mental Health Team], and to Out-reach [social support team]. Then the three of us have the referral and we all tend to see them the next morning, and we then try to come to some sort of plan together. That's the way it should work"*.

However, when queried if the three teams come together in some form of case conference, the interviewee replied:

*"No not really. It's difficult. We tend to see them quite quickly whereas CMHT, unless it's urgent have a 3 day [waiting list]. So we often see them first and it depends on the drug use, because there may be drug use and mental health problems but the drug use might just be that they smoke cannabis at the weekend and have a severe mental illness, and those cases would be taken over primarily by CMHT but those with a very heavy drug use, we take on and then do the mental health assessment. And then we liaise with them and make them aware, and if we have concerns then we will speak to the psychiatrist ourselves. So it depends on the individual and their risk and their needs really but sometimes we're all working with the same person..."***(detox staff, ID 52)**

## Discussion

The health behaviours, clinical and demographic background of prisoners make an important contribution to health in prisons. However, the environment, the regime, and the organisational culture are likely to be more important [1,2,12]. Medication practices are a key indicator of and contributor to the therapeutic prison environment. They have particular relevance in light of findings that approximately 20% of male and 50% of female prisoners take some form of psychotropic medication [13], and that the taking of mood-modifying medicines such as minor tranquillisers and other psychotropic medication provides support and encourages patient engagement. An important element of the later is to encourage patients to participate in daily decisions about treatment which in turn is perceived to be a key part of their recovery [7].

This study found a tension in the standards and nature of official policy concerning mental health and what is happening on the ground. Prison policy espouses the goal of delivering mental health services to prisoners that provide 'effective through-care that responds quickly and seamlessly to their changing needs' [2], yet a common theme to emerge in the descriptions of both inmates and prison staff indicated *discontinuity *in medication treatment received on entering prison. Such findings are contrary to the purported goal of seeking to normalise mental health care in prisons to equate with the norms, values and practices existing in the wider community. Delays, stoppages or changes to medication were noted to be the underlying causes of confusion, anxiety and distress reported by half of prisoners interviewed. At a time when prisoners are perceived to be in a particularly vulnerable state and experience the loss of normal social support, such actions should be recognised as representing the removal of a prop. Restrictions to self-medicate further limit individuals' opportunities to engage in self-medication and management that are generally available to them in community settings.

The role of staff in providing high quality mental health care has been highlighted by HM Chief Inspector of Prisons review [5. This report stresses the importance of relationships between prison staff and prisoners, and warns of the danger of staff failing to recognise the impact that entry to prison for the first time can have. Notwithstanding the deficits associated with local prisons (the high turnover of the prison population, overcrowding, inadequate resources to provide purposeful activity etc) and the difficulties encountered by nursing and medical staff working in an environment in which 'healthcare culture is influenced by traditional attitudes, with an emphasis on security [1], the importance of doctor-patient communication has been suggested as playing an important role in securing effective treatment outcomes. Indeed, it has been suggested that patients have multiple needs which fail to be voiced due to doctors misinterpreting what their patient's agendas actually are [14]. The findings of this study confirm this to be particularly so in prison settings where pre-conceived notions of prisoners' objectives, the limited amount of time available to conduct patient interviews, and doctors' attempts to 'fit in' with established prison practice [1]. The latter often resulted in frequently rushed, impersonal consultations in which little attention appears to be afforded to prisoners' concerns and little interest shown in discussing treatment options. The dysfunctional nature of such interactions both promote and perpetuate prisoners' views of health care as being another form of discipline, whilst they themselves are generally perceived as 'problematic' or 'malingerers' [15].

## Conclusion

One risk that requires managing in prison settings is the obtaining and use of prescription drugs ....for which there is no medical justification. Accounts of staff and observations carried out in the course of the present study suggested that a proportion of prisoners may attempt to deceive healthcare staff in order to obtain prescriptions. An unfortunate corollary of this is that prisoners as a group are typecast as being untrustworthy and manipulative; such labelling consigning those who present with genuine mental health problems (as indicated by formal screening and previous community management) to greater suffering, loss of control, deterioration in mental health state and risk. Whilst some ....prison healthcare staff have a role to play in deciding on prescription changes or encouraging self medication practices which are conducive to patients' routines and needs, it is likely that even those performing to the best of their ability are likely to be constrained by organisational, environmental and cultural factors which currently restrict or pose barriers to introducing standards of service that are commonplace in the wider community and to which the Prison Service aspires. The distribution of in-possession medication and control over self- medication is to be promoted to those who would benefit. This is already accepted working practice in some establishments; risk assessment and the establishment of appropriate protocols having been incorporated into working practices. The supply of prescription drugs which may be subject to subsequent misuse remains a risk for prison staff that needs to be managed. However, findings suggest that attention needs to be given to removing institutional barriers, and changing professional practices and interactions with inmates with mental health problems in a way that is more therapeutically orientated. The limited opportunity for consultation during rushed reception procedures, the availability of appropriately trained and experienced medical staff at such times, restrictions to patient contact due to the prison regime [16], and the technological improvements to modernise prison records and IT systems are just a few examples that undermine the effective care of prisoners. In the absence of the organisational changes required to effect more flexible working practices [17] and afford healthcare staff the time they need to 'unpack' prisoners' health matters and engage in dialogue that addresses their concerns, this paternalistic approach to restricting prescribed medication will continue to the detriment of those in greatest need and hamper progress towards the goal of ensuring that prisoners receive an equivalent standard of care to that more widely available in the NHS.

Despite evidence of the scale of mental disorder among prisoners and an acute awareness of long established deficits in the standard of prison healthcare, much of the progress towards addressing the inherent vulnerability of such prisoners appears to have been slow in its implementation limited. Current moves to introduce mental health in-reach teams and dedicated detox. facilities are an important step to improve patient access to specialist services. Nonetheless, their effectiveness is likely to be undermined if there is no change in routine practices and a greater awareness of the need and opportunities for prisoners to take personal responsibility for their treatment. The latter is relevant for developing therapeutic relationships and the nurturing of ideologies that support the interests of a more therapeutic regime [18].

## Competing interests

The authors declare that they have no competing interests.

## Authors' contributions

RB designed and modified the qualitative study and undertook the field work and drafted the manuscript, AR designed the study, assisted with data analysis and interpretation and drafted the manuscript, JS designed the larger study within which this project was based and contributed comments to the manuscript.

## Appendix 1

Superscript information

^1. ^Local prisons accommodate prisoners who have been remanded in custody awaiting trial, those who have been convicted but not yet sentenced, and those serving short sentences.

^2. ^Adult male prisoners in England and Wales are classified into one of four security categories A, B, C or D based on the likelihood of escape and the risk to the public if they did escape; Category A (Cat. A) being the most secure. Unless given Cat. A status, women and YOs are not categorised other than requiring either closed or open conditions; closed conditions being designed to prevent escape.

^3. ^The prisons taking part in this study were all participating in the Care of At Risk Prisoners project which was part of the wider Safer Locals Programme of environmental and procedural changes aimed at improving the detection and management of prisoners at risk of suicide and self-harm.

^4. ^The F2052SH system of management applies to prisoners considered to be at risk of self-harm or suicide. F2052SH refers to the designated form used to record details of the care programme initiated. This process was superceded by ACCT.

^5. ^As a replacement for the F2052SH system, the trial of ACCT (Assessment, Care in Custody and Teamwork) was instigated in 2004 and became policy in all prisons in England and Wales in 2007.^6. ^In-reach teams are made up of multidisciplinary staff initially intended to assist in the management of prisoners with severe and enduring mental illness.

^7. ^HCOs - Healthcare officers - prison officers who have undertaken specialist training in health care; some of whom may have a nursing qualification.

## Supplementary Material

Additional file 1 **Table S1 - Outline of the mental health problems experienced by prisoner participants**. Additional table and related information. Click here for file
