# Supplementary material for: Medication management and practices in prison for people with mental health problems: a qualitative study
Source: Int J Ment Health Syst. 2009 Oct 20;3:24. doi: 10.1186/1752-4458-3-24 (PMC2770990; doi:10.1186/1752-4458-3-24)
Supplement: Additional file 1 — Table S1 - Outline of the mental health problems experienced by prisoner participants. Additional table and related information. [file 1752-4458-3-24-S1.DOC]

Table S1: Outline of the mental health problems experienced by prisoner participants

| Time point for data collection | T1 | T2 |
| --- | --- | --- |
| No. of prisoner participants | 20 | 19 |
| (A) - Number of pps. with history of mental illness / sub. misuse | 18 | 18 |
| Number of (A) pps. with a history of mood disorder | 10 | 8 |
| Number of (A) pps. with a history of psychotic illness | 6 | 9 |
| Number of (A) pps. only to have history of substance misuse | 2 | 1 |
| Number of (A) pps. with history of self-harm / suicide | 13 | 14 |
| Number of (A) pps. placed on F2052SH / ACCT supervision | 16 | 14 |

The following table provides details of the range of mental disorder suffered by the purposive sample participating in the current study.

**APPENDIX 1: Details of participants at Time T1**

| Prisoners |  |  |
| --- | --- | --- |
| Gender | Male  Female | 12  8 |
| Age | <25 years  <35 years  <45 years  <55 years | 5  10  3  2 |
| Main Offence | Drug related  Acquisitive  Violence  Miscellaneous | 4  7  7  2 |
| Time in prison | <1 month  <3 months  <6 months  ≥6 months | 6  7  2  5 |
| Experience of  the F2052SH system | Yes  No | 17  3 |
| History of  mental illness | Yes  No | 15  5 |
| History of  Self-harm | Yes  No | 13  7 |
| Drug / alcohol  problem | Yes  No | 13  7 |
| Prison staff |  |  |
| Gender | Male  Female | 25  17 |
| Role | Chaplain  Detox. staff  Doctor  Healthcare nurses / HCOs  In-reach staff  Social Worker  Prison officer  Probation  Psychiatrist  Psychology  Suicide Prev. Coordinator | 3  3  2  10  3  1  12  1  2  1  4 |

**APPENDIX 1 ctd.: Details of participants at Time T2**

| Prisoners |  |  |
| --- | --- | --- |
| Gender | Male  Female | 15  4 |
| Age | <25 years  <35 years  <45 years  <55 years | 8  7  4  0 |
| Main Offence | Drug related  Acquisitive  Violence  Miscellaneous | 2  5  7  5 |
| Time in prison | <1 month  <3 months  <6 months  ≥6 months | 6  7  2  4 |
| Experience of  ACCT | Yes  No | 15  4 |
| History of  mental illness | Yes  No | 14  5 |
| History of  Self-harm | Yes  No | 14  5 |
| Drug / alcohol  problem | Yes  No | 12  7 |
| Prison staff |  |  |
| Gender | Male  Female | 18  11 |
| Role | Detox. staff  Doctors / Psychiatrists  Healthcare nurses / HCOs  In-reach staff  Prison officer  Suicide Prev. Coordinator  Social worker  Occupational therapy | 3  3  6  5  7  3  1  1 |
